# Quality Evaluation of Fair-Trade Cocoa Beans from Different Origins Using Portable Near-Infrared Spectroscopy (NIRS)

**DOI:** 10.3390/foods12010004

**Published:** 2022-12-20

**Authors:** Matteo Forte, Sarah Currò, Davy Van de Walle, Koen Dewettinck, Massimo Mirisola, Luca Fasolato, Paolo Carletti

**Affiliations:** 1 Dipartimento di Agronomia, Animali, Alimenti, Risorse Naturali e Ambiente, Università degli Studi di Padova, Viale dell’Università 16, 35020 Legnaro, PD, Italy; 2 Dipartimento di Biomedicina Comparata e Alimentazione, Università degli Studi di Padova, Viale dell’Università 16, 35020 Legnaro, PD, Italy; 3 Food Structure and Function Research Group, Department of Food Technology, Safety and Health, Faculty of Bioscience Engineering, Ghent University 653, 9000 Ghent, Belgium; 4 Dipartimento di Medicina Animale, Produzioni e Salute, Università degli Studi di Padova, Viale dell’Università 16, 35020 Legnaro, PD, Italy

**Keywords:** *Theobroma cacao* L., dry matter, chemometrics, fermentation index, protein content

## Abstract

Determining cocoa bean quality is crucial for many players in the international supply chain. However, actual methods rely on a cut test protocol, which is limited by its subjective nature, or on time-consuming, expensive and destructive wet-chemistry laboratory procedures. In this context, the application of near infrared (NIR) spectroscopy, particularly with the recent developments of portable NIR spectrometers, may represent a valuable solution for providing a cocoa beans’ quality profile, in a rapid, non-destructive, and reliable way. Monitored parameters in this work were dry matter (DM), ash, shell, fat, protein, total polyphenols, fermentation index (FI), titratable acidity (TA) and pH. Different chemometric analyses were performed on the spectral data and calibration models were developed using modified partial least squares regression. Prediction equations were validated using a fivefold cross-validation and a comparison between the different prediction performances for the portable and benchtop NIR spectrometers was provided. The NIRS benchtop instrument provided better performance of quantification considering the whole than the portable device, showing excellent prediction capability in protein and DM quantification. On the other hand, the NIRS portable device, although showing lower but valuable performance of prediction, can represent an appealing alternative to benchtop instruments for food business operators, being applicable in the field.

## 1. Introduction

With an ancient history starting in the Preclassic period (1200–400 B.C.) with consumption among the Olmec and other pre-Colombian populations of the Americas [1], cocoa is now a ubiquitous food. Cocoa appears today in many different forms, mainly in chocolate, with consumption averaging around 8 kg per person per annum in many European countries [2]. The top four countries, which account for nearly 65% of the total world chocolate production, are the USA, Germany, Switzerland, and Belgium, which in terms of retail sales reach respectively USD 20, USD 10, USD 14, and USD 12 billion per year [3]. The consumption of cocoa and cocoa-based products is of great interest both for the highly appreciated sensorial profile and for the possible beneficial health effects which are being studied in current times [4,5].

Cocoa beans represent the essential raw material for chocolate, and they are obtained from the *Theobroma cacao* L. tree, which is almost exclusively cultivated in tropical/developing countries, where it represents a source of export earnings both at the level of families, communities, and nations [6]. Africa covers more than 75% of the world’s total cocoa beans production and Côte d’Ivoire is the largest cocoa bean exporter in the world, with more than USD four billion exported in 2020, followed by Ghana and Ecuador.

Once the cacao pods have been harvested, they must undergo post-harvest operations on farms and plantations before becoming the so-called cocoa beans, which are then traded in the international market and processed into final industrial products. The post-harvest processing comprise pod opening and removal of beans from the pod, bean-pulp mass fermentation and bean drying. In this sequence, the fermentation constitutes an essential critical step for the development of desired flavor attributes of the commercial cocoa beans. In the further processing, cocoa beans are roasted, cracked and ground to give a powdery mass from which fat is expressed [7] and the release of fat ultimately leads to a liquid-like ingredient, namely cocoa liquor. Additionally, many processes are implemented in the chocolate industry that originate several products with different forms and functionalities.

Fermentation and drying constitute key farm(er)-based unit operations with strong influences in the final quality of cocoa beans and subsequent products [8].

Since 2010, approximately 4 million tons of cocoa beans have been produced annually around the world [9], and the three biggest importers are the Netherlands, Germany, and USA, with product worth 2,375,923, 1,209,366 and 1,026,931 USD imported in 2020, respectively (Sources: ITC calculations based on UN COMTRADE and ITC statistics).

The socio-economic importance and international interest towards cocoa beans are intelligible as an estimated five million farming households depend on cacao as a cash crop, and 70 per cent of cocoa is produced by smallholders living on less than USD 2 per day and relying on cocoa for 60 to 90 per cent of their income [9].

Cocoa beans are divided worldwide between “fine or flavor” (mainly Criollo and Trinitario) and “bulk” (mainly Forastero) varieties, with a common belief that fine and flavor cacao varieties receive significant price premiums in international markets. However, recent studies show how post-harvest processing has a central role in causing heterogeneity in cocoa prices, independently of the variety grown [10]. This is because although the primary factors influencing the quality attributes of cocoa beans are the cocoa tree cultivar and genotype, it is well-established that the agronomic and environmental conditions together with the harvest and post-harvest steps are crucial elements in the determination of the final quality of commercial cocoa beans [11,12,13,14,15].

Partially fermented or unfermented beans are prone to bitterness and astringency with poor chocolate flavor and aroma [2]. Moreover, an appropriate drying process will reduce the beans’ water content of 55% to around 7% [16], preventing fermentation from continuing uncontrolled, slowing the development of molds that could give rise to unwanted, unpleasant flavors and equilibrating the beans acidity that would otherwise be excessive in the final products [17,18].

Although fermentation is considered as the “core stage” of the cocoa transformation process from seeds to chocolate, it is currently performed mostly by small third-world producers in an empirical way, with little or no technification, without control in processing conditions, originating cocoa batches of low and heterogeneous quality [13].

Chocolate and chocolate-based products sell in a very competitive market, where quality is crucial, and value is enormous. It should be clear that if the quality of cocoa beans is poor, final products will suffer this deficiency as well, and the whole industry sector will be affected [19].

Most of the existing commercial standards for cocoa beans base their quality requirements on the results of the cut test, or on sensory estimation by trained panels (Aculey et al., 2010). The cut test consists of cutting cocoa beans lengthwise, observing the number of defective beans. The ISO 1114 states that both halves of each bean shall be visually examined, and the result for each kind of defect shall be expressed as a percentage of the 300 beans examined. The ISO also defines nine categories of defects: those related to poor fermentation (slaty and violet/purple beans) and those being indicators of high FFA levels, poor flavor and/or other contaminants (bean clusters, broken beans, smoky beans, moldy beans, germinated beans, flat beans, insect-damaged/infested beans) [20].

On the one hand, the cut test is limited by its subjective nature and does not represent a sufficiently reliable methodology for a comprehensive description of the main quality contributors [21]. On the other hand, laboratory methodologies are often demanding in terms of time and cost effectiveness, which can be critical factors, and they are mainly based on destructive determinations. Moreover, in cocoa producing countries the availability of laboratory infrastructures is poor [22].

In this context, the application of near infrared (NIR) spectroscopy, particularly with the recent developments of portable NIR spectrometers, may represent a valuable solution for providing a cocoa beans’ quality profile, in a rapid, non-destructive, and reliable way. This analytical technique could be useful to both cocoa bean producers, mostly in the developing countries, and processors, mostly in the developed countries, alike.

Many researchers have already investigated quality parameters of cocoa beans through NIR spectroscopy quantifying, for instance, fat, sugars, proteins, moisture, pH and titratable acidity, polyphenols, and other volatile and non-volatile compounds [23]. Even the assessment of the authenticity of cocoa powder has been studied by identifying the country of origin of raw materials, varietal purity, or the presence of adulterants [24].

It must be noted, however, that most of the studies that successfully predicted cocoa beans’ quality parameters through NIR spectroscopy mainly analyzed samples which had been purposely subjected to different degrees of fermentation, e.g., analyzing the beans at different days during the fermentation process. Doing so, the samples are not in the status in which importers/exporters normally trade them in the international market, fully fermented and dried, therefore this might not be a representative “working condition” for these actors in the cocoa supply chain. Moreover, only cocoa bean samples coming from one or few countries are most often utilized in the previous studies, hence limiting the variability that can be included in the NIR prediction model. Finally, most of the studies present in literature utilize benchtop NIR instruments, which have some notable practical disadvantages if compared with the more recent portable NIR spectrometers, which are recently being considered in the literature [25,26].

On this basis, our study aimed to predict some quality parameters of commercial cocoa bean samples using portable NIR spectrometers, also in comparison with a benchtop spectrometer, on both whole and ground samples. Cocoa beans were provided by an Italian fair-trade importer and all the samples have been produced to meet internationally accepted merchantable quality standards: well-fermented and dry. The ultimate purpose of the study was, therefore, to evaluate the possibility of using portable NIR spectrometers in commercial contexts, while also assessing the practicability of using NIR spectroscopy on whole fermented coca beans to rapidly predict main quality parameters.

## 2. Materials and Methods

### 2.1. Samples

Fifty-six samples from commercially available cocoa beans have been provided by Altromercato Impresa Sociale Soc. Coop. (Via 9. Crispi, Bolzano, Italy), a major importer of fair-trade products in Italy in 2021. Thirty-three of them came from Africa while the remaining twenty-three came from South America. All the samples have been produced to meet internationally accepted merchantable quality standards i.e., well-fermented and dry, free from smoky beans and abnormal or foreign odors, free from evidence of adulteration, reasonably free from living insects, virtually free from broken beans, pieces of shell, and foreign matter. African countries included Togo, Uganda, Madagascar, and Sierra Leone, while American beans came from Honduras, Ecuador, Perú, Dominican Republic, Nicaragua, and Venezuela (Supplementary Table S1). Although it was not possible to obtain accurate information on the beans’ variety for each sample, documents reported that only Trinitario and Forastero varieties were utilized.

The beans were shipped in 25-ton containers which roughly corresponds to 360 bags (70 kg of cocoa beans/bag). Around 4 kg of cocoa beans from each container were sampled as representative for the 33% of the total bags’ number. Of these, 200 g of cocoa beans were randomly taken for analysis and stored at −20 °C in plastic bags until assay in a LGPv 8420 MediLine refrigerator (Liebherr, Kirchdorf an der Iller, Germany).

### 2.2. Beans Peeling and Grinding

Three random aliquots of 20 g of dry and nitrogen frozen cocoa beans from each sample were de-husked by hand. The peels and nibs were carefully collected and weighted to determine average shell percentage on a 4 digits balance (Adventurer model ARRV70, OHAUS, Parsippany, NJ, USA). About 100 g of nibs (de-husked cocoa beans) were ground in a multi-purpose grinder for 45 s (3 intervals of 15 s with 10 s pause), then sifted on a 0.5 mm sieve. Before grinding, the beans were frozen with liquid nitrogen to make them brittle and avoid becoming a mash. The obtained cocoa powder for each sample was stored at −20 °C in the dark prior to the following analyses.

### 2.3. Spectral Data Acquisition

Spectral data acquisition was performed both on whole cocoa beans and on de-husked cocoa bean powder. About 100 g of randomly chosen whole cocoa beans from each sample were scanned with a portable instrument (PoliSPEC-NIR, ITPhotonics, Breganze, Italy) and with the benchtop instrument (FOSS DS−2500 scanning monochromator FossNIR-System, Hillerød, Denmark). Both NIR data acquisitions were performed in reflectance mode, with the following parameters:

− FOSS DS-2500: scanning monochromator covering a range of 850–2500 nm at 0.5 nm intervals. Scans were performed using a slurry cup with quartz window of about a 12.6 cm^2^ area.
− PoliSPEC-NIR: covering a range of 900–1680 nm at 2 nm intervals. Spectral data measurements were performed through a round scanning window (3.2 cm^2^) placed in direct contact with the sample surface. Each spectrum was obtained by averaging 3 data acquisitions.

### 2.4. Chemical Analyses

Unless otherwise specified, analyses were performed according to official methods of analysis (AOAC, 2016). All chemical analyses were performed in triplicate on peeled and ground cocoa beans.

#### 2.4.1. Dry Matter

Dry matter is measured as subtraction of the moisture content measured using a gravimetric method based on AOAC method 931.04 [21]. Hereto, approximately 2 g of powder sample were dried at 101–103 °C to constant weight in a forced-air electric oven (UF55 Plus, Memmert, Schwabach, Germany). After the drying process was completed, the samples were immediately closed with glass lids to avoid exposure and stored in desiccators for one hour to equilibrate samples towards ambient temperature [27]. The moisture content was expressed as average percentage (%) based on loss in weight of three independent samples.

#### 2.4.2. Ash

For the measurement of ashes, the sample was charred on a plate and placed in a muffle furnace (Gefran Model 1200; Gefran Spa, Brescia, Italy) at 550 °C (AOAC 972.15A). Ash content was expressed as weight percentage (%).

#### 2.4.3. Fat Content

The fat content was measured by extraction with petroleum ether [21] in a TE-188 Soxhlet lipid extractor (model SOXTEC 255 Tecator-Foss Analytical, Hillerød, Denmark) with the following parameters: 60 min boiling, 50 min washing, 15 min drying. Fat content was expressed as weight percentage (%).

#### 2.4.4. Total Protein Content

Protein determination was carried out by the Kjeldahl method, as described in AOAC 2016 (method 970.22) (model Kjeltec 2300-Foss Analytical). The protein content was calculated from the concentration of total nitrogen by applying a conversion factor of 6.25.

#### 2.4.5. Total Phenolic Content

The total phenolic content was determined according to the colorimetric method of Folin–Ciocalteu [28]. Samples were defatted using the Soxhlet method (AOAC 963.15). Defatted powder (0.05 g) was added to 10 mL of a methanol-water (70:30 *v*/*v*) mixture at room temperature and stirred for 45 min. After centrifugation, 0.1 mL of solution was mixed with 3 mL of distilled water and 0.5 mL Folin–Ciocalteu reagent. The mixture was stored for 3 min after which 1 mL of aqueous Na_2_CO_3_ (200 g L^−1^) was added. The mixture was allowed to stand for 20 min at 40 °C and the total polyphenols were determined by spectrophotometry at 765 nm (spectrophotometer model Cary 60 UV-Vis Agilent Technologies Stevens Creek Blvd. Santa Clara, CA, USA). The standard curve was prepared using 0, 50, 100, 150, 200 and 250 mg L^−1^ solutions of gallic acid in methanol. Total phenol values were expressed in terms of gallic acid equivalents (mg g^−1^ of dry fat-free mass) [29]. The analyses were performed in triplicate.

#### 2.4.6. Fermentation Index

Fermentation index (FI) corresponds to the color change within the bean cotyledons during fermentation. This change is due to the decreasing anthocyanin content as beans progress through fermentation [30]. A 50 mg sample of previously prepared cocoa powder was weighed and mixed with 5 mL MeOH:HCl (97:3 *v*/*v*). Samples were extracted at 4 °C for 16–18 h, centrifuged for 5 min at 3500× *g*, and the clear supernatant was collected. Absorbance of the supernatant was read at wavelengths 460 nm and 530 nm using UV-VIS spectrophotometer (model Cary 60 UV-Vis Agilent Technologies Stevens Creek Blvd. Santa Clara, CA, USA). All the measurements were performed in triplicate.

The FI was obtained by calculating the ratio of the absorbance at 460 nm and 530 nm (FI = A_460_/A_530_). Values greater than 1 are considered as well-fermented, while less than 1 as under-fermented beans [31,32]. However, it must be noted that this accounts for the Forastero variety and with some precautions for the Trinitario variety (which can contain both purple and white beans). Criollo beans do not contain anthocyanin pigments, therefore FI cannot be used to describe the fermentation level for this variety. In our study, both Trinitario and Forastero beans were used, but white beans were always absent.

#### 2.4.7. pH and Titratable Acidity

Cocoa powder (5 g) was mixed with 100 mL hot water (100 °C), stirred, and allowed to stand for 30 min. After 30 min, when the suspension was cooled up to 25 °C, it was centrifuged for 10 min at 5000 rpm and vacuum filtered through Whatman No. 4 paper filter according to AOAC 2006 methods 970.21 (pH) and 942.15 (potentiometric titration) [21], AOAC Section 42.104 (16th Ed. 1995) [21,32,33,34,35].

The pH of the filtered solution was measured with a pH-meter model PC 80 + DHS (XS Instruments, Carpi, Italy) and then 25 mL aliquots of the same solution were titrated to pH 8.1 with 0.05 M NaOH. All data were measured in triplicate. Titratable acidity results are expressed as mMol NaOH/100 g powder [34] or % acetic acid [21].

It is important to note that this procedure was not for quantifying the actual pH of the cocoa bean itself, but rather to measure the acidity derived from bean acids diffusing into water; it is useful for comparison between the pH of solutions produced by different beans [30].

### 2.5. Wavelenght Selection and Chemometric Analyses

Spectral chemometric analyses were performed using firstly the wavelength selection and secondly the full spectra collected. Wavelengths selection was carried out through the interval partial least-square (iPLS) [36] and through the principal component regression (PCR) [37] by using R software version 3.2.5 (R Core Team, Auckland, New Zealand, 2016) and WinISI software (Infrasoft International, Port Matilda, PA, USA), respectively. In particular, the iPLS was carried out applying the forward mode, in which the full spectrum was subdivided in 30 intervals that are successively included in the analysis: the first step calculated 30 models (one for each interval) that were tested using the cross-validation; the interval which provides the lowest model root-mean-square error of cross-validation were selected as most informative. The selected intervals were calculated per each parameter investigated and used for the following modelling. The PCR is based on the identification of the principal factors variance among spectral absorbance data through the principal component analysis [38]. Wavelengths selection was performed on the spectra acquired with FOSS DS-2500 on cocoa powder.

The second approach considered the use of the full spectrum and mathematical treatment as reported by several authors [39,40,41] in foods for chemical prediction purpose. This procedure takes advantage of the mathematical treatment as multiplicative correction (MSC) of the dispersion used to correct the problems of dispersed light in reflectance spectroscopy or the spectra normalization using standard normal variation (SNV) and first or second derivatives often used to remove the deviation and slope of the baseline in the spectrum [42]. This approach was applied to spectra acquired with both instruments, on both whole and ground cocoa beans.

The calibration models were performed using the Modified PLS (MPLS) regression on wavelength selected and on full spectra, whereas PCR was applied on full spectra (WinISI software, Infrasoft International, Port Matilda, PA, USA). Prediction equations were validated using a 5-fold cross-validation. Samples with a predicted value that differed more than 2.5 SD from the reference value (T-statistics) were considered outliers and removed from the dataset. Several combinations of scatter corrections (NONE, no correction; SNV_DT, standard normal variate and detrending; MSC, multiplicative scatter correction) and derivative mathematical treatments (0,0,1,1; 1,4,4,1; 2,5,5,1; where the first digit is the number of the derivative, the second is the gap over which the derivative is calculated, the third is the number of data points in the first smoothing and the fourth is the number of data points in the second smoothing) were tested. The performances of the prediction models were evaluated based on the number of the standard error of calibration (SEC), cross-validation (SECV), the coefficient of determination of cross-validation (R^2^cv) and the ratio performance to deviation of cross-validation (RPDcv) calculated as the ratio between SD and SECV [43]. Predictions were considered excellent when R^2^ was greater than 0.91, good when R^2^ ranged from 0.82 to 0.90, approximate when R^2^ was between 0.66 and 0.81, and poor when R^2^ was less than 0.66 [44]. Prediction models with RPD greater than 2.5 were considered adequate for analytical purposes [45], whereas prediction models with RPD smaller than 1.5 were considered unsatisfactory [44].

## 3. Results and Discussion

### 3.1. Chemical Properties

Shell content was on average 13.25% (Table 1), with minimum and maximum values (11.13% and 18.34%, respectively) in line with those reported in the literature (12–20%) [46,47]. Although the shell provides protection to the nib from mold and insects infestations, the shell content should be as low as possible (10–14%) because it has very little commercial value for the cocoa processor: it is removed during cocoa bean processing and it mainly constitutes a waste material [48].

Dry matter was on average 94.51%, with a minimum of 93.30%. These values correspond to an average moisture content of 5.49% and maximum of 6.70%, which are mainly below the optimal commercial levels of 6.5–8.0% as reported in CAOBISCO/ECA/FCC [19] but are in line with data found in the literature [49]. Moisture is a parameter that depends on storage conditions: since storage conditions of the studied samples varied, this may have affected the final moisture levels.

The average ash content of 2.99% found in our samples was in line with data reported in the literature [48,50]. With regards to fat content, which is the most abundant macronutrient in cocoa beans, only one sample presented a value below 40 g/100 g (i.e., 36.96 g/100 g), while the average fat content was 44.72 g/100 g. These data are in line with other studies [21,50]. African cocoa beans have generally higher fat content than American beans [16], but this was not observable in our set of samples. However, according to literature, the fat content can vary greatly from values of about 40 g/100 g to values of 57–58 g/100 g depending on different factors such as: genotype, plant age, growing practices, fermentation, drying processes and environmental conditions [51,52].

FI is one of the most used parameters for determining the degree of fermentation of cocoa beans as an indirect measure of the anthocyanin content [29,35]. In our case study, 22 out of 56 samples had a FI slightly below 1, with a minimum value of 0.57, which would indicate a low fermentation degree. The maximum value was 2.24 and the average was 1.29. The coefficient of variation for this parameter was particularly high (38.43%). Since the FI is an indirect measurement of anthocyanin content, the high dispersion of data might be due to factors other than solely the fermentation degree. It has been reported that different hybrids or genotypes have different pigments and that phenolic compounds are quantitatively affected by cocoa growth conditions (microclimate and position of pods on the tree) [29].

The TPC in the dried fat-free mass of our samples exhibited a wide variation, ranging from 32.58 to 98.04 mg/g dry defatted powder. In fermented beans, TPC should be approximately 5% in the dried fat-free mass, and values above 10% are considered a sign of a bad fermentation [53]. The average value of TPC in our samples was 56.42 mg/g dry defatted powder (equals to 5.6%) that would indicate well-fermented beans. Moreover, few samples showed values close to 10%. Overall, the values are in line with those reported in Anyidoho, et al. [54] and Djikeng, et al. [55].

In dried cocoa beans, a high degree of acidity is usually associated with a pH of 5.0 or less [19]. Some studies report that beans of higher pH (5.5–5.8) are considered unfermented, with a low fermentation index, and result in chocolates with high astringency [32], while beans of lower pH (4.75–5.19) are considered as well-fermented. Other studies report that pH of 5–6 is considered good for flavor development, and cocoa beans with pH below 4.5 are not accepted by cocoa bean processers because they show low levels of flavor precursors, and high acidic-derived products [35]. The pH can still be considered as a good indicator of fermentation as higher pH correlates to a lower fermentation degree [16] and an “international acceptable range” of 5.00–5.55 for dried cocoa beans [56] can be considered as a valid reference. In our case study, cocoa beans had an average pH of 5.58 with a minimum of 4.84. This describes a situation of well fermented samples.

The titratable acidity value is often associated with the beans’ pH. The present results confirm an overall good fermentation of the samples with an average titratable acidity of 17.19 mmol NaOH/100 g powder, in line with data reported in the literature [57,58].

Overall, this set of samples included many variation factors (e.g., genetic variety, crop, fermentation and drying conditions, transport, and storage) giving rise to high coefficients of variation in most of the studied parameters [59].

### 3.2. Spectral Characteristics of Cocoa Samples

**Figure 1:**
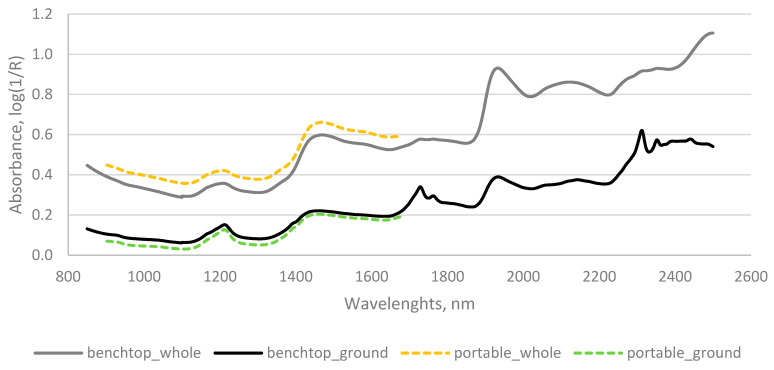
NIR spectra (mean) of whole (gray) and ground (black) cocoa beans acquired with benchtop spectrometer (FOSS DS 2500) and NIR spectra (mean) of whole (yellow) and ground (green) cocoa beans acquired with portable NIR spectrometer (PoliSPEC-NIR).

Figure 1 is representative for average NIR spectra of cocoa beans samples obtained by FOSS DS 2500. The spectra show high similarity with spectra found in the literature [21,27,50,51]. Since cocoa beans contain about 50% of fat (Table 1), absorption spectra are dominated by signals derived from C=O and CH_2_ groups [49]. The absorptions around 1930 nm are caused by the second overtone vibration of ester C=O and O–H asymmetric stretching [49,60]. Caporaso, et al. [61] reported that wavelength of 1919 nm has been attributed to the C=O stretching second overtone in the carbonyl groups (–CO_2_H or CONH) but this absorption band is very close to 1923 nm, which is assigned to the O–H group of water and therefore it might be influenced by this group.

The combination vibrations of CH_2_ stretch and CH_2_ deformation appear around 2320 nm. Moreover, the absorption at 1744 nm has been previously assigned to C–H stretch first overtone (CH_2_) of lipids, and the CH_2_ group also absorbs at 1725 nm, due to the C–H stretch first overtone [61]. Similar wavelength values (i.e., 1750 nm and 1730 nm), associated with first overtones of symmetric and anti-symmetric C–H stretch vibration (CH_2_-groups), are reported by Krahmer et al. [49].

Fat content is also related to the absorption bands visible around 1200 nm, as reported by Hayati et al. [27]. The authors also argued that the bands in the wavelength regions of 1460–1490 nm and 1920–1980 nm are most likely related to moisture content (O–H bonds). However, absorbances around 1450 nm have been attributed to carbonyl groups (e.g., ketones and aldehydes) as well as O–H polymeric groups, which can be due to complex carbohydrates, and the region between 1400 nm and 1440 nm has also been attributed to aliphatic alcohols and phenols [61].

Absorbance around 1490 nm has been attributed in the literature to several possible chemical bond vibrations, including N–H stretch first overtone and O–H stretch first overtone, thus indicating amides or compounds such as cellulose [61]. Accordingly, Krahmer et al. [49] reported that first overtones of intermolecular H-bridges and stretch vibrations of amidic NH-groups can be observed in the region of 1400 to 1500 nm and the corresponding combination of two amides can be found around 2130 nm.

Barbin et al. [50] associated the broad peaks around 1190, 1460 and 1950 nm with O–H, C–H, N–H stretch first and second overtones and combination bands that can be attributed to water absorption and protein changes.

Peaks around 1215 nm are visible and are associated with –CH=CH second overtone [23] and even C–H stretching second overtone (–CH_3_ or –CH_2_) of carbohydrates is associated with this wavelength [61].

The absorbance at 2057 nm indicates an N–H stretch/amide 1st combination band, which has been attributed to protein, while the peaks at 2145 and 2313 nm have been tentatively attributed to C–H deformation and C–H deformation and C–H bend second overtones respectively, both indicating lipids [61].

### 3.3. Calibration Models for Cocoa Beans Quality

Variable selection is generally applied in the multivariate analysis to extract the most informative region, removing redundant information. However, among the approaches tested in this study, a lower prediction was observed for the PCR than the MPLS approach as observed in the study of Xie et al. [37]. In detail, in the present study, the PCR showed poor performance of prediction for all traits investigated (see Supplementary Table S2).

Comparing the performance of prediction using the MPLS between full and iPLS selected spectra, it was observed that among the eight parameters, the best prediction was achieved using the full spectra for seven of them (see Supplementary Table S3). The iPLS wavelength selection had a better performance in the fat prediction (R^2^cv of 0.86 and RPD of 2.88) that did not differ substantially from the prediction obtained using the whole spectrum (900–1680 nm; R^2^cv = 0.83 and RPD = 2.43).

The results of prediction performance for the benchtop (NIR FOSS DS 2500) and the portable (PoliSPEC-NIR) spectrometers are presented in Table 2 and Table 3, which describe data obtained from whole cocoa beans and peeled-ground cocoa beans, respectively.

Generally, most of the cocoa studies were performed on ground cocoa to reduce the effects of the physical sample properties on spectra collection [24]. Indeed, for both NIRS devices, the best performances of prediction were observed on ground sample, probably due to the enhanced homogeneity of the samples characterized by a similarity in the particles size and in a more compacted powder that affects the scattering of light.

In this study, spectra corrections by mathematical treatments to remove irrelevant data such as noise and background information were evaluated. In particular, SNV and MSC were used as pre-processed methods to remove the influence of solid particle size and the surface scattering; moreover, the methods above are mainly recognized as the best mathematical treatment in the equation models developed for whole cocoa. The SNV_DT and MSC treatments improved the prediction accuracy for some quality parameters of both whole and ground cocoa bean samples, while for other parameters raw spectra gave the best results. This was in line with Barbin et al. [50] who found no considerable improvement of the predictive ability when comparing different pre-processing methods with the original raw data. Indeed, Barbin et al. [50] stated that since the complexity of the models was similar to that obtained with the original data, it is feasible to use the raw spectra to build prediction models for both whole beans and ground cocoa samples.

Moreover, to evaluate the performance of technologies on the market, the whole spectrum was considered to perform the prediction equations, although some researchers suggest that selection of spectral intervals could lead to higher prediction performances [35,62]. All the predictions performed against whole bean sample can be considered as approximate to poor [44] with the highest capability achieved for DM (R^2^cv = 0.72; RPDcv = 1.86) for the benchtop and for pH (R^2^cv = 0.70; RPDcv = 1.83) with portable device (Table 2). In general, the minor prediction capability in whole cocoa beans compared to the ground sample has been confirmed also in the study of Hernández-Hernández et al. [63], in which the poor performance of chemical predictions was attributed to the shell that reflects the incident light hindering the interaction with internal constituents. Although predictions on whole cocoa beans were not adequate for quantitative purposes, they could represent a fast approach for food business operators to sort cocoa beans towards a specific transformation according to high or low value. Moreover, at germplasm banks and breeding programs, a rapid whole cocoa analysis reduces the time required for the shell removing (usually carried out by hand in the laboratory), suggesting NIRS devices are capable to identify functional genotypes to improve qualitative aspects in cocoa products [63].

Excellent performance was obtained in ground cocoa for protein content (R^2^cv = 0.91; RPDcv = 3.40) and very good prediction was achieved for DM (R^2^cv = 0.90; RPDcv = 3.20), ash (R^2^cv = 0.89; RPDcv = 2.98), pH (R^2^cv = 0.88; RPDcv = 2.96) and TA (R^2^cv = 0.86; RPDcv = 2.70) using the NIR FOSS DS 2500 spectrometer (850–2500 nm) (Table 3). The PoliSPEC-NIR spectrometer (900–1680 nm) had the best predicting performances for fat content (R^2^cv = 0.82; RPDcv = 2.34) in ground samples; whereas, for the other traits, the portable device showed lower performances compared to the benchtop (Table 3).

To deeper investigate if the divergences between the devices might depend on the different spectral range used, a further prediction equation was performed for the benchtop using the same spectral range (900–1680 nm, every 2 nm) of the portable tool. In the comparison with the performance obtained considering the whole spectrum, a greater performance of predictions was observed for ash (R^2^cv = 0.90; RPDcv = 3.20), protein (R^2^cv = 0.93; RPDcv = 3.84), DM (R^2^cv = 0.94; RPDcv = 4.16), and lipids (R^2^cv = 0.83; RPDcv = 2.43). However, although the TPC remained unpredictable, an increment was observed in the new prediction equation (R^2^cv = 0.46; RPDcv = 1.37). Although a good predictive capability was maintained, lower performance prediction was observed for TA (R^2^cv = 0.85; RPDcv = 2.60) and pH (R^2^cv = 0.82; RPDcv = 2.34).

**Figure 2:**
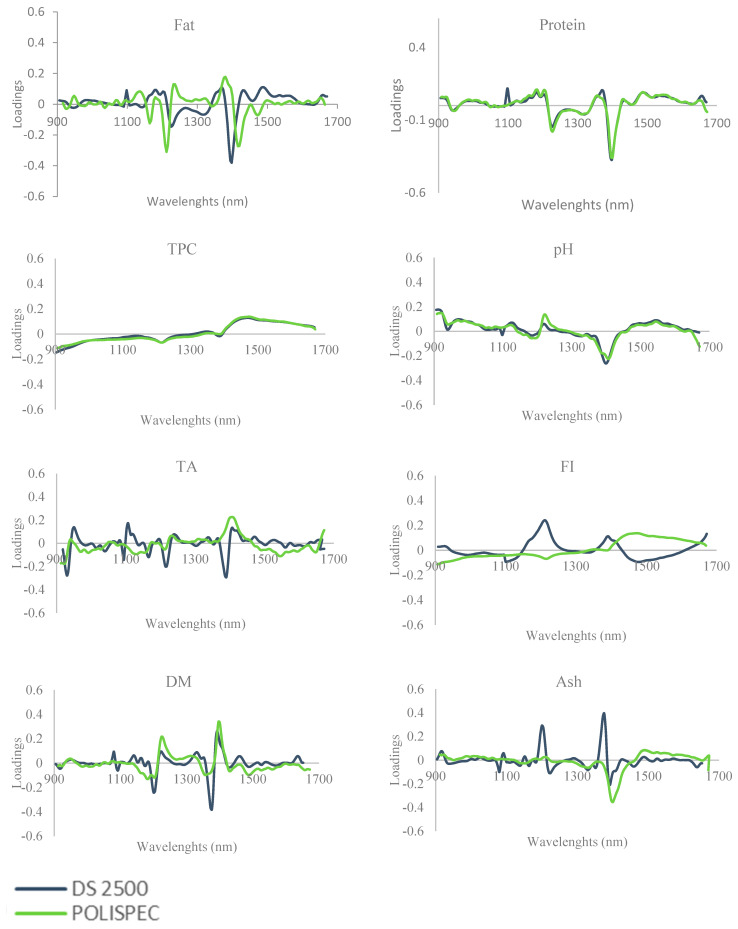
Loadings for the first principal component of fat, protein, pH, titratable acidity (TA), total phenolic compound (TPC), fermentation index (FI), dry matter (DM), and ash for NIR spectra of the ground cocoa samples for DS 2500 (blue line) and PoliSPEC NIR (green line).

Thus, to comprehend the origin of the performance divergences between devices, the component loadings were developed for each tool to assess and compare the interactions between wavelengths and functional groups (Figure 2). The loading plots permit to better understand which wavelengths are more informative for a specific trait variability, showing the range which is mostly considered to develop the model. A strong similarity between portable and benchtop devices were overall observed for chemical parameters directly quantified.

In particular, although the same ranges and performance of prediction (R^2^cv = 0.83) were obtained in both devices for lipid loading plot, the highest loadings were observed in the spectral region between the 1212 and 1232 nm and 1368 and 1398 nm for portable and benchtop, respectively.

Such association between those range and lipid variability has been confirmed by [64] in cereal food products. Similar patterns for the protein loading plot were observed between the two devices; however, the high loadings observed between 1200 to 1400 nm were related to C–H second overtone and N–H stretching first overtone of protein, respectively [65,66]. Moreover, a high loading was observed around 1100 nm exclusively for the benchtop device; this is probably due to the higher sensitivity of the device that is reflected in the best performance of prediction (R^2^cv = 0.93) the range 1100–1400 nm being considered as an essential spectral region for the protein quantification analysis [67].

A comparable loading plot was also observed for pH in which the highest trait variability was explained by the 910 [68] and 1398 nm for both devices. Divergences in titratable acidity loading patterns were found; however, the most informative wavelengths (930–950; 1106; 1390–1400 nm) are related to the second combination region of the carboxylic acids [69]. The loading plot of DM showed notable peaks between 1200–1224 and 1373–1394 nm, mainly related to the water [69].

Ash being an inorganic matter cannot be directly detected by NIRS; its amount is indirectly measured by the association with organic bonds, thus the loadings plot and the highest variability observed for ash is 1200 nm and 1376 nm for the benchtop, and 1396 nm for the portable device account for other organic components. Otherwise, loadings plots observed for TPC and FI were not strictly related to a specific spectrum range, probably due to the lower variability collected with the samples considered. In general, the performance divergences between the two NIRS devices could be explained by the difference in the detector equipment; in detail, the semiconductors included in portable (PoliSPEC-NIR) and benchtop (NIR FOSS DS 2500) devices are Indium gallium arsenide (InGaAs) and silicon lead sulfide, respectively, which affect the spectral response and the prediction capability Lin, et al. [70].

In our study, the accuracy of prediction for both FI and TPC was not satisfactory for any of the instruments and for both whole and ground cocoa bean samples. The influence of variable fermentation degrees of cocoa samples can be crucial in the prediction of FI and TPC, which are strictly related to the fermentation level of cocoa beans. Sunoj, Igathinathane and Visvanathan [32] showed how factors such as pod storage duration (before the fermentation process), and fermentation time, had a significant effect on the fermentation index, which was seen to increase together with the increment of these two parameters. The authors argued that these parameters are indirectly affected by the samples’ chemical composition, thus the accuracy of prediction models are generally lower than those reported for major components. The reason might fall on the fact that our samples included only commercial cocoa beans which were supposed to be well-fermented, although with some natural variations, thus reducing the variability for the TPC and FI. Moreover, there could have been a negative influence of lipid absorbances in the models for TPC: fat has been indicated as a disturbance factor as beans with higher relative fat content have lower non-fat solids, where polyphenols are concentrated [61].

Although the FI was not correctly predicted by the constructed models, the estimations in ground samples of parameters related to correct fermentation such as pH and TA were approximative and good with the portable and benchtop devices, respectively, in line with previous results [25,49]. This method could provide a rapid and low-cost multiparametric analysis for cocoa evaluation. Portable instruments are usually less expensive than benchtop solutions (about a fifth) [71], and the cost of analyses are mainly related to the development and upgrade of calibration curves. Moreover, compared to wet analyses, through the application of spectrometric methods the cost of the analytical determination is drastically reduced as the number of examined samples increases.

The presented prediction models might be the basis for an overall cocoa bean quality evaluation based on NIR spectra. However, despite the presented parameters being good indicators of cocoa bean quality, a grading classification of cocoa beans’ was beyond the scope of the present work, as it would require the investigation of other indicators, also related to the sensorial profile of the beans, as reported in previous studies on cocoa quality indexes (CQI) [72,73].

## 4. Conclusions

The results of this paper demonstrated that NIRS portable and benchtop devices coupled with chemometrics methods could be adopted for the chemical evaluation of commercial cocoa beans. The performances of predictions are affected by the presence of shell and the sample particle sizes of cocoa beans. The current study has successfully demonstrated that NIR, as a nondestructive analytical method, can be considered as rapid and reliable option to traditional methods to quantify lipids, protein, pH, titratable acidity, dry matter and ash in cocoa ground beans.

The NIRS benchtop instrument provided better performance of quantification considering the whole (800–2500 nm) and the reduced spectrum (900–1680 nm) than the portable device. Variable selection through iPLS or PCR did not improve prediction models compared to full spectra analyses. Benchtop instrument showed excellent prediction capability in DM (R^2^cv = 0.94), protein (R^2^cv = 0.93) and ash (R^2^cv = 0.90), whereas lipids (R^2^cv = 0.83), TA (R^2^cv = 0.86) and pH (R^2^cv = 0.88) were well predicted on ground beans considering wavelengths between 900–1680 nm. Those results indicate that models developed for benchtop devices are applicable for cocoa quality control as an excellent option to substitute conventional methods.

On the other hand, the NIRS portable device showed lower but valuable performance of prediction than benchtop spectrometer. The prediction obtained for handheld device represents an appealing strategy for food business operators to apply in the field to control and check the product in every phase of trade and transportation, and also to segregate whole cocoa beans targeted to a specific transformation in different supply chains.

Based on these results, further studies including a wider variability of fermentation phases, cocoa bean varieties and origins as well as additional production steps of the cocoa supply chain could be investigated to support the fair-trade cocoa sector.

## Figures and Tables

**Table 1:** Descriptive statistics of cocoa beans: SD = standard deviation; CV = coefficient of variation; TPC = total phenolic component; TA = titratable acidity; FI = fermentation index; DM = dry matter.

	Minimum	Maximum	Mean	SD	CV (%)
Shell (%)	11.13	18.34	13.25	1.54	11.59
Fat (%)	36.96	48.39	44.72	1.94	4.35
Protein (%)	8.32	15.43	13.85	1.13	8.14
TPC (mg/g dry defatted powder)	32.58	98.04	56.42	13.32	23.60
pH	4.84	6.47	5.58	0.36	6.50
TA (mMol NaOH/100 g powder)	8.20	26.81	17.19	4.22	24.52
FI (A460/A530)	0.57	2.24	1.29	0.49	38.43
DM (%)	93.30	95.76	94.51	0.59	0.62
Ash (%)	2.34	3.66	2.99	0.30	10.10

**Table 2:** Fitting statistics of prediction models for whole cocoa traits developed using cross-validation results for benchtop (NIR FOSS DS 2500) and portable (PoliSPEC-NIR) NIR-spectrometers.

FOSS DS 2500										
Math Treatment		Constituent	N	Mean	SD	SEcal	R^2^cal	SEcv	R^2^cv	RPD
NONE	1441	Fat	52	2.48	0.26	0.13	0.77	0.15	0.69	1.78
NONE	2551	Protein	51	0.77	0.09	0.04	0.81	0.06	0.66	1.71
SNV_DET	2551	TPC	54	3.04	0.66	0.61	0.15	0.67	0.03	0.98
NONE	0011	pH	54	5.60	0.36	0.21	0.65	0.24	0.58	1.52
MSC	0011	TA	53	16.81	3.99	2.73	0.53	2.98	0.46	1.34
MSC	2551	FI	55	0.07	0.03	0.02	0.25	0.03	0.07	1.03
NONE	1441	DM	55	94.49	0.57	0.26	0.80	0.31	0.72	1.86
MSC	2551	Ash	54	0.16	0.03	0.01	0.87	0.02	0.51	1.43
**PoliSPEC-NIR**										
**Math Treatment**		**Constituent**	**N**	**Mean**	**SD**	**SEcal**	**R^2^cal**	**SEcv**	**R^2^cv**	**RPD**
MSC	1441	Fat	54	2.45	0.27	0.18	0.57	0.21	0.38	1.28
SNV_DET	0011	Protein	50	0.78	0.10	0.04	0.83	0.07	0.56	1.49
MSC	2551	TPC	48	3.04	0.60	0.22	0.87	0.40	0.56	1.51
MSC	0011	pH	50	5.56	0.37	0.18	0.76	0.20	0.70	1.83
MSC	1441	TA	55	17.26	4.22	2.77	0.57	3.03	0.48	1.39
NONE	0011	FI	55	0.07	0.03	0.02	0.66	0.02	0.58	1.55
MSC	0011	DM	49	94.50	0.54	0.26	0.77	0.32	0.66	1.72
SNV_DET	0011	Ash	53	0.16	0.03	0.02	0.66	0.02	0.45	1.36

NONE = no correction; SNV_DET = SNV and detrend; MSC = multiplicative scatter correction; SD = standard deviation of reference data selected; SEcal = standard error in calibration; R^2^cal = coefficient of determination of calibration; SEcv = standard error in cross-validation; R^2^cv = coefficient of determination of cross-validation. TPC = total phenolic compound; TA = titratable acidity; FI = fermentation index; DM = dry matter.

**Table 3:** Fitting statistics of prediction models for ground cocoa traits developed using cross-validation results for benchtop (NIR FOSS DS 2500) and portable (PoliSpec NIR) NIR-spectrometers.

FOSS DS 2500										
Math Treatment		Constituent	N	Mean	SD	SEcal	R^2^cal	SEcv	R^2^cv	RPD
NONE	1441	Fat	56	2.45	0.27	0.11	0.84	0.13	0.76	2.11
SNV_DET	0011	Protein	54	0.77	0.11	0.02	0.95	0.03	0.91	3.40
NONE	2551	TPC	55	3.04	0.65	0.51	0.40	0.59	0.16	1.10
MSC	0011	pH	50	5.57	0.37	0.08	0.95	0.13	0.88	2.96
NONE	1441	TA	52	16.69	3.86	0.80	0.96	1.43	0.86	2.70
NONE	1441	FI	55	1.29	0.50	0.27	0.70	0.38	0.42	1.31
MSC	0011	DM	56	94.51	0.59	0.15	0.94	0.18	0.90	3.20
MSC	0011	Ash	50	0.16	0.03	0.01	0.90	0.01	0.89	2.98
**PoliSPEC-NIR**										
**Math Treatment**		**Constituent**	**N**	**Mean**	**SD**	**SEcal**	**R^2^cal**	**SEcv**	**R^2^cv**	**RPD**
NONE	2551	Fat	52	2.45	0.27	0.09	0.88	0.12	0.82	2.34
NONE	0011	Protein	53	0.77	0.11	0.04	0.84	0.05	0.79	2.17
MSC	0011	TPC	55	3.04	0.65	0.50	0.42	0.57	0.23	1.14
NONE	1441	pH	53	5.56	0.36	0.11	0.90	0.18	0.74	1.98
NONE	0011	TA	51	17.23	4.07	1.14	0.92	1.76	0.81	2.32
NONE	0011	FI	56	1.29	0.49	0.41	0.33	0.42	0.26	1.17
NONE	0011	DM	54	94.54	0.59	0.25	0.81	0.27	0.79	2.17
NONE	1441	Ash	54	0.16	0.03	0.01	0.89	0.01	0.76	2.08

NONE = no correction; SNV_DET = SNV and detrend; MSC = multiplicative scatter correction; SD = standard deviation of reference data selected; SEcal = standard error in calibration; R^2^cal = coefficient of determination of calibration; SEcv = standard error in cross-validation; R^2^cv = coefficient of determination of cross-validation. TPC = total phenolic compound; TA = titratable acidity; FI = fermentation index; DM = dry matter.

## Data Availability

All related data and methods are presented in this paper. Additional inquiries should be addressed to the corresponding author.

## Supplementary Materials

The following supporting information can be downloaded at: https://www.mdpi.com/article/10.3390/foods12010004/s1, Table S1: Cocoa origin of different commercial lot analysed in this study Table S2: Fitting statistics of prediction models for ground cocoa traits developed using full spectra and principal component regression (PCR) and cross-validation results for benchtop (NIR FOSS DS 2500); Table S3. Fitting statistics of prediction models for ground cocoa traits developed using selected wavelengths through the interval PLS (iPLS) and cross-validation results for benchtop (NIR FOSS DS 2500).
