# Supplementary material for: Quality Evaluation of Fair-Trade Cocoa Beans from Different Origins Using Portable Near-Infrared Spectroscopy (NIRS)
Source: Foods. 2022 Dec 20;12(1):4. doi: 10.3390/foods12010004 (PMC9818779; doi:10.3390/foods12010004)
Supplement: Supplementary file 1 [file foods-12-00004-s001.zip › foods-2085157-supplementary.pdf]

Table S1. Cocoa origin of different commercial lot analysed in this study.

SAMPLE	Commercial label	Origin
**1**	TCLU 6447817	Togo
**2**	TCKU 6003395	Ecuador
**3**	GCNU 4779442	Togo
**4**	GCNU 4791601	Togo
**5**	GCNU 4791582	Togo
**6**	CAIU 7176858	Togo
**7**	TCKU 7334740	Perù
**8**	MSCU 5732757	Togo
**9**	MSCU 5004140	Togo
**10**	GCNU 4780504	Togo
**11**	ACLU 9786610	Togo
**12**	GCNU 4732086	Togo
**13**	GCNU 4779442	Togo
**14**	DFSU 6281608	Togo
**15**	MSCU 5563050	Togo
**16**	MSCU 7865379	Togo
**17**	TGBU 5781302	Togo
**18**	FFAU 1707460	Togo
**19**	MEDU 4134886	Togo
**20**	TCKU 6876563	Nicaragua
**21**	20/045	Togo
**22**	20/030	Sierra Leone
**23**	90/20	Ecuador
**24**	20515-3	Africa
**25**	60111	Dominican Republic
**26**	20/01-A	Perù
**27**	97676A	Venezuela
**28**	2368/2021	Uganda
**29**	94060V	Africa "Maina"
**30**	200530-000	Uganda
**31**	97318	Dominican Republic
**32**	1141963	Madagascar
**33**	1181672	Honduras
**34**	C2019-01/VC01946	Perù
**35**	FBLU 0076159	Uganda
**36**	2378/2021	Perù
**37**	910597	Africa
**38**	115.2167A	Ecuador
**39**	2362/2021	Africa
**40**	AGS-345	Venezuela
**41**	PTC 200023-02	Sierra Leone
**42**	HLXU 8490101	Ecuador

**43**	22	Ecuador
**44**	TCLU 4723773	Togo
**45**	99/20	Ecuador
**46**	20515-1	Africa
**47**	VCU 1815 A	Perù
**48**	AGS-351	Venezuela
**49**	DN-19003870	Africa
**50**	BEAU 4992555	Perù
**51**	TRHU 4573123	Perù
**52**	3210601418	Ecuador
**53**	175	Ecuador
**54**	CAIU71766858	Togo
**55**	HASU5184268	Perù
**56**	2551/2021	Africa

Table S2. Fitting statistics of prediction models for ground cocoa traits developed using full spectra and principal component regression (PCR) and cross-validation results for benchtop (NIR FOSS DS 2500).

Math Treatment		Constituent	N	Mean	SD	SEcal	R^2cal	SEcv	R^2cv	RPD
NONE	0011	Fat	56	2.45	0.27	0.15	0.72	0.15	0.70	1.83
NONE	0011	Protein	54	0.77	0.10	0.04	0.81	0.05	0.77	2.09
SNV_DET	1441	TPC	53	2.99	0.62	0.51	0.32	0.52	0.31	1.19
SNV_DET	1441	pH	55	5.57	0.36	0.27	0.45	0.28	0.43	1.32
SNV_DET	1441	TA	55	17.24	4.23	3.31	0.39	3.34	0.38	1.27
SNV_DET	1441	FI	56	1.29	0.49	0.47	0.09	0.46	0.16	1.09
NONE	0011	DM	56	94.51	0.59	0.25	0.82	0.26	0.81	2.28
NONE	0011	Ash	56	0.16	0.03	0.02	0.68	0.02	0.66	1.67

NONE = no correction; SNV_DET = SNV and detrend; SD = standard deviation of reference data selected; SEcal= standard error in calibration; R^2cal = coefficient of determination of calibration; SEcv = standard error in cross-validation; R^2cv = coefficient of determination of cross-validation. TPC = total phenolic compound; TA = tirtatable acidity; FI = fermentation index; DM = dry matter.

Table S3. Fitting statistics of prediction models for ground cocoa traits developed using selected wavelengths through the interval PLS (iPLS) and cross-validation results for benchtop (NIR FOSS DS 2500).

Math Treatment		Constituent	N	Mean	SD	SEcal	R^2cal	SEcv	R^2cv	RPD
NONE	0011	Fat	51	2.47	0.26	0.09	0.89	0.09	0.86	2.88
NONE	0011	Protein	52	0.76	0.11	0.03	0.93	0.04	0.90	2.75
NONE	0011	TPC	54	3.04	0.65	0.62	0.13	0.65	0.08	1.00
NONE	0011	pH	54	5.57	0.37	0.17	0.79	0.21	0.69	1.76
NONE	0011	TA	54	17.27	4.13	2.24	0.71	2.85	0.52	1.45
NONE	0011	FI	56	1.28	0.50	0.33	0.55	0.39	0.36	1.28
NONE	0011	DM	55	94.50	0.59	0.19	0.89	0.20	0.88	2.95
NONE	0011	Ash	54	0.16	0.03	0.01	0.83	0.01	0.77	2.07

NONE = no correction; SD = standard deviation of reference data selected; SEcal= standard error in calibration; R^2cal = coefficient of determination of calibration; SEcv = standard error in cross-validation; R^2cv = coefficient of determination of cross-validation. TPC = total phenolic compound; TA = tirtatable acidity; FI = fermentation index; DM = dry matter.
